# Twelve-year natural history of a gastric adenocarcinoma of fundic gland type

**DOI:** 10.1007/s12328-016-0680-5

**Published:** 2016-09-13

**Authors:** Yoshinori Sato, Takashi Fujino, Akira Kasagawa, Ryo Morita, Shun-ichiro Ozawa, Yasumasa Matsuo, Tadateru Maehata, Hiroshi Yasuda, Masayuki Takagi, Fumio Itoh

**Affiliations:** 1 Division of Gastroenterology and Hepatology, Department of Internal Medicine, St Marianna University School of Medicine, Sugao Street 2-16-1, Miyamae-ku, Kawasaki, Kanagawa 216-8511 Japan; 2 Department of Pathology, St Marianna University School of Medicine, Kawasaki, Kanagawa Japan; 3 Division of Research and Development for Minimally Invasive Treatment, Cancer Center, Keio University, School of Medicine, Tokyo, Japan

**Keywords:** Gastric adenocarcinoma of fundic gland type (GAFG), Endoscopic submucosal dissection (ESD), Natural history

## Abstract

A 77-year-old woman underwent an upper gastrointestinal (UGI) endoscopy screening examination, and a 10-mm reddish, submucosal tumor-like lesion was found on the posterior wall of the fornix. Biopsy was performed, but there was no evidence of malignancy, so annual follow-up by UGI endoscopy was decided upon. After 12 years, examination of another biopsy specimen revealed an adenocarcinoma of the fundic gland type. There had been no significant change in the size or shape of the lesion over the long follow-up period. Endoscopic submucosal dissection (ESD) was performed, and en bloc resection was achieved. Histopathologically, the tumor appeared as a flat elevated lesion measuring 11 × 10 mm. It was composed of irregularly shaped glands and invaded the submucosa up to 300 µm. Immunohistochemical examination involving specific antibodies to pepsinogen I, MIST-1, MUC6, and H^+^/K^+^-ATPase confirmed the fundic gland differentiation of the irregularly shaped glands together with a very low Ki-67 labeling index. Thus, gastric adenocarcinoma of the fundic gland type (GAFG) was diagnosed. Four years have passed since the ESD, and there has been no recurrence. To the best of our knowledge, this is the first report of the long-term natural history of GAFG. Over the 12 years, no morphologic changes were observed; the tumor remained within the submucosal layer. Our observations in this case strengthen the notion that GAFG is a specific type of gastric adenocarcinoma of low-grade malignancy.

## Introduction

Gastric adenocarcinoma of the fundic gland type (GAFG, chief cell predominant type) was, in 2010, proposed by Ueyama et al. as a new histologic type of gastric cancer [1]. GAFG is immunohistologically positive for pepsinogen I, a gastric fundic gland cell marker, and H^+^/K^+^-ATPase, a parietal cell marker. There are clear clinicopathological differences between GAFG and common gastric adenocarcinoma [1, 2]. GAFG generally presents as a well-differentiated adenocarcinoma comprising tumor cells with a low degree of atypia and resembling non-neoplastic oxyntic glandular cells; the degree of malignancy is low, and the prognosis is good. However, the progression of these tumors remains unclear because high-grade malignancy lesions, with some infiltrating the subserosal layer, have been reported [3]. We describe a case of GAFG that we monitored endoscopically and histopathologically for 12 years after the initial diagnosis. This case is informative because it sheds light on the natural history of GAFG.

## Case report

**Fig. 1:**
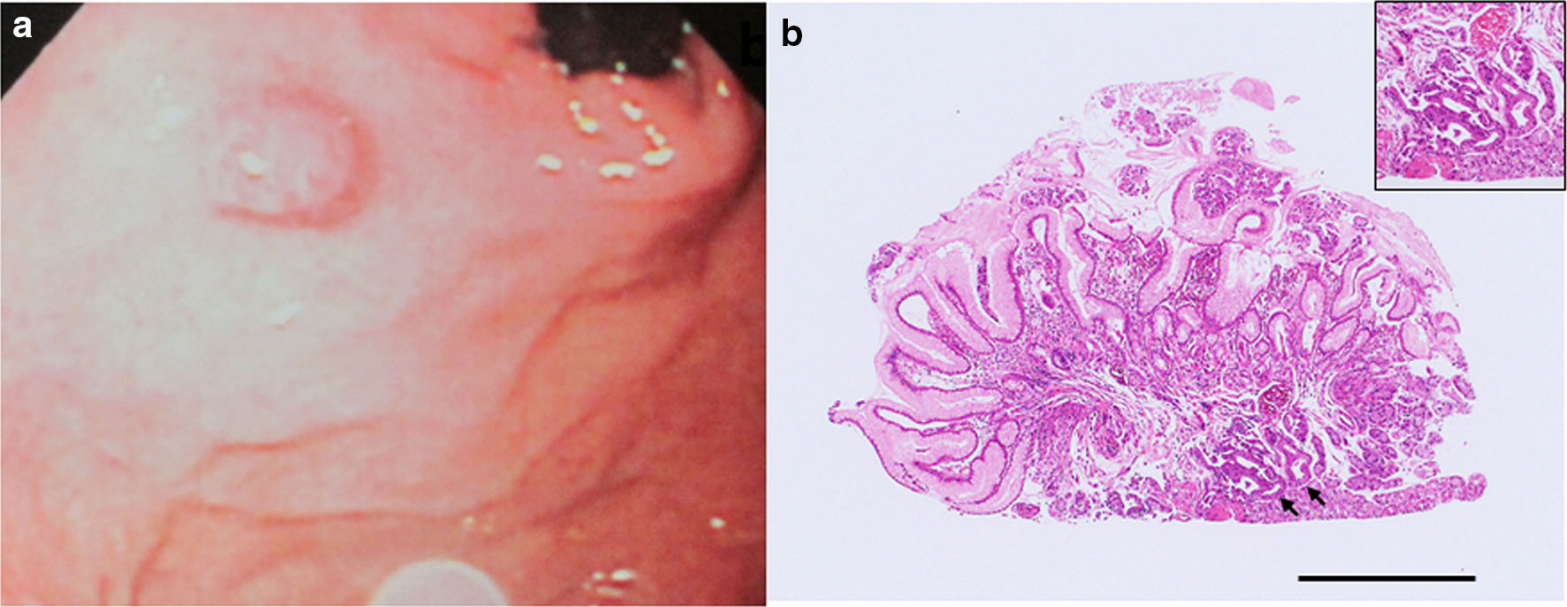
Endoscopic appearance of the lesion and histologic features of the biopsy specimen obtained upon the initial endoscopic examination (H&E stain). **a** The initial endoscopic examination revealed a *reddish*, 10-mm, elevated lesion on the posterior wall of the fornix (shown here in the polaroid photograph obtained at the time). **b** Non-neoplastic glands had been detected in the initial biopsy specimen, but upon re-examination of the original specimen after endoscopic treatment, we noted atypical glands corresponding to GAFG (*black arrows*). *Black bar* 1 mm. *Inset* high magnification view of the GAFG

**Fig. 2:**
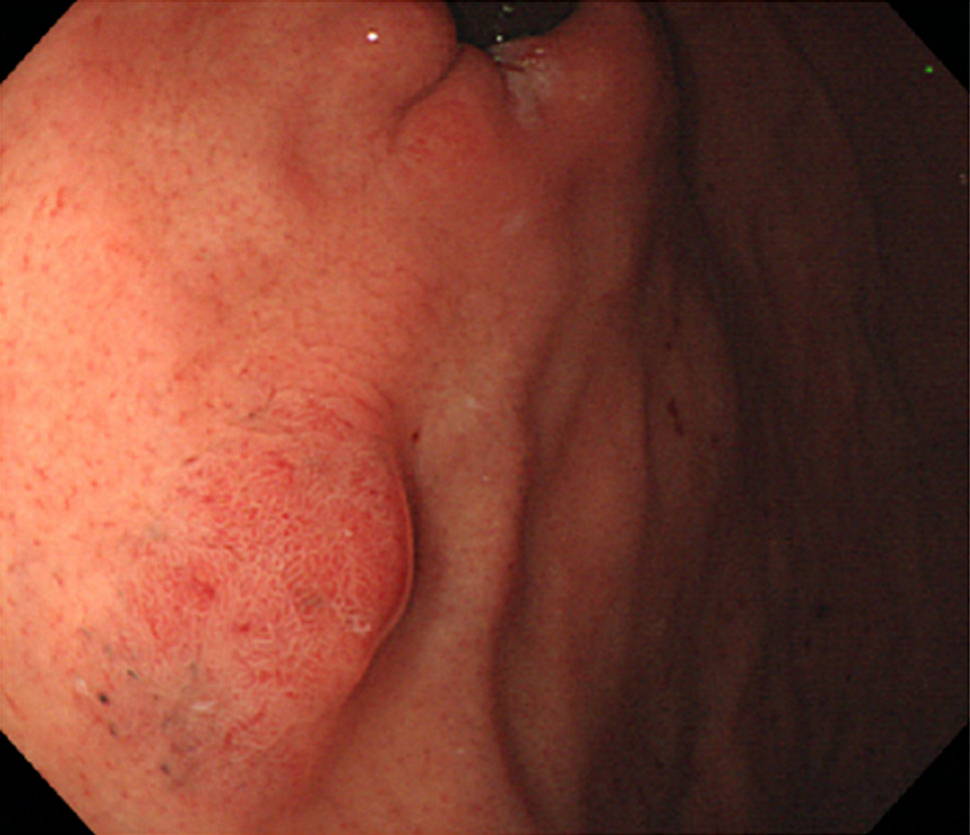
Endoscopic appearance of the lesion at 8 years later after initial endoscopy. Endoscopic examination performed 8 years later revealed a smooth, *reddish*, submucosal tumor-like elevated lesion, 10 mm in diameter. *Black* pigmentation and dilated vessels with a branching architecture were seen on the surface of the tumor

The patient was a 77-year-old woman who underwent a routine upper gastrointestinal (UGI) endoscopy screening at our hospital. She was in good health and had no family history of gastrointestinal cancer. The examination revealed an elevated, 10-mm-diameter, smooth, reddish, submucosal tumor-like lesion in the posterior wall of the fornix (Fig. 1a). Biopsy was performed, and a non-neoplastic lesion was diagnosed (Fig. 1b), so we decided to simply monitor the case by means of annual UGI endoscopy. A biopsy specimen was obtained almost every year at the time of endoscopy. At 8 years, the lesion remained elevated, but it had flattened slightly in comparison to the morphology noted at the initial examination, probably due to the effect of repeated biopsies. There was no obvious change in the size of the tumor, but dilated vessels with a branching architecture and black pigmentation were seen on the surface (Fig. 2). No malignancy was detected in biopsy specimens obtained subsequently. However, at 12 years, histologic examination of the biopsy specimen suggested GAFG. The lesion was still 10 mm, and it had retained its smooth, reddish appearance, but there was a protrusion. There was no evidence of submucosal invasion, so we decided to perform endoscopic resection.

Upon admission for endoscopic treatment, the patient’s general health was good, and according to laboratory tests, there was no elevation in tumor markers, including carcinoembryonic antigen (CEA), carbohydrate antigen (CA) 19-9, and alpha-fetoprotein (AFP), and the serum was negative for *Helicobacter pylori* antibody (<3 U/ml, Anti-*Helicobacter pylori* IgG antibody, EIA kit. SRL Stock company).

**Fig. 3:**
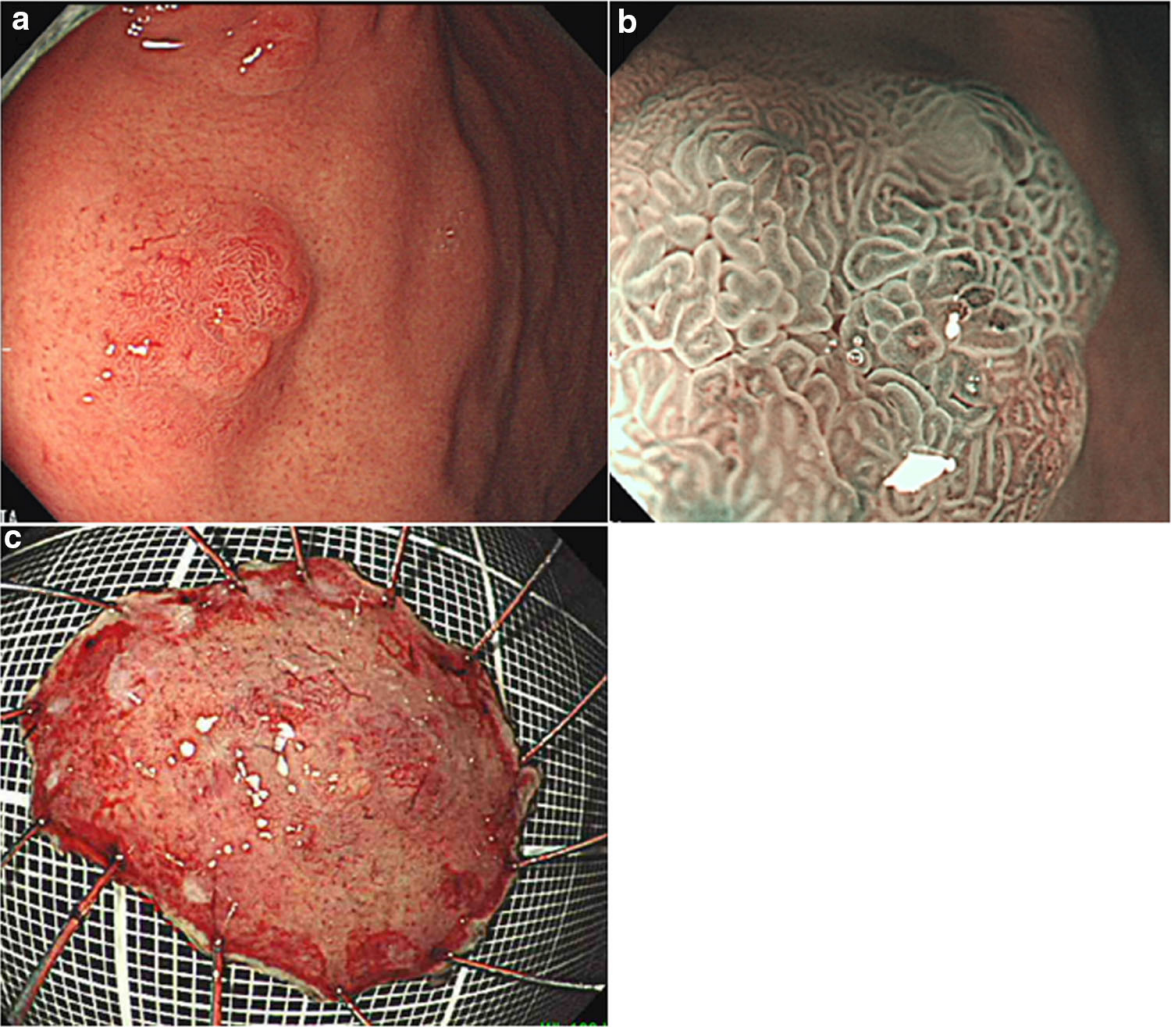
Endoscopic examination performed before endoscopic submucosal dissection confirmed the presence of (**a**) a well-circumscribed, 10-mm, *reddish*, smooth, elevated lesion in the posterior wall of the fornix. The tumor was soft, and there was no evidence of expanding appearance. There was no clear line of demarcation. Dilated vessels exhibiting a branching architecture were observed on the surface. Atrophic change was not seen in the surrounding mucosa. **b** Magnifying endoscopy with narrow band imaging revealed a regular MV pattern and a regular MS pattern without a demarcation line. **c** The resected tissue measured 28 × 22 mm, and the flat elevated lesion measured 11 × 10 mm

**Fig. 4:**
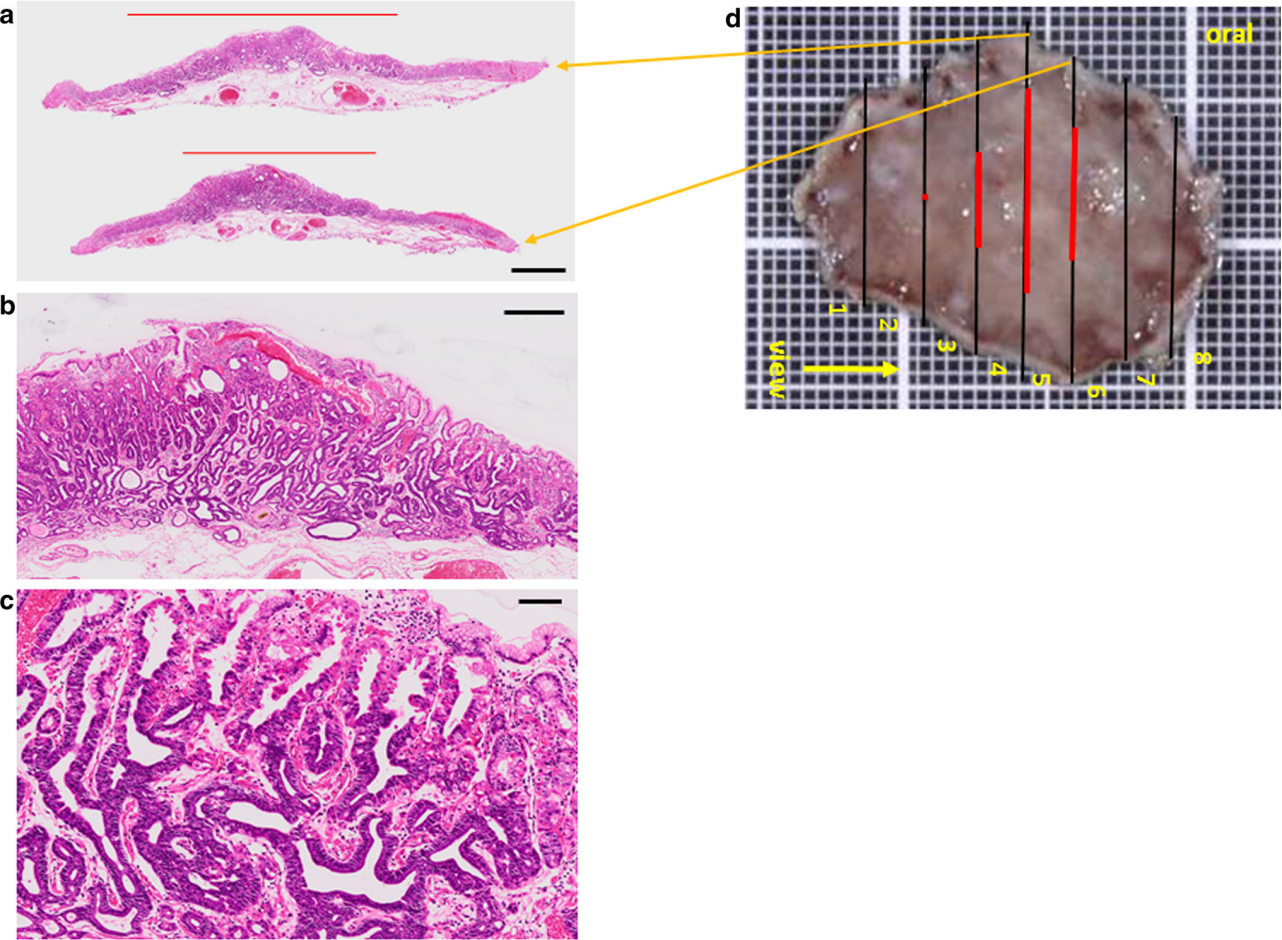
Histopathologic features of the ESD specimen. **a** Histologic slides under loupe magnification showing the spread of the adenocarcinoma. *Black bar* 2 mm. **b** Low magnification image shows irregularly shaped neoplastic glands lying mainly in the lower portion of the oxyntic mucosa. *Black bar* 500 μm. **c** High magnification image. Note the irregularly shaped glands consisting of two types of atypical glandular cells, those with basophilic cytoplasm, and those with eosinophilic cytoplasm. *Black bar* 200 μm. **d** Spread of the carcinoma mapped on the surgical specimen. *Black line* *cut line*, *Red line* spread of the carcinoma. *Orange arrows* from the *cut lines* point to the respective loupe images (**a**)

**Fig. 5:**
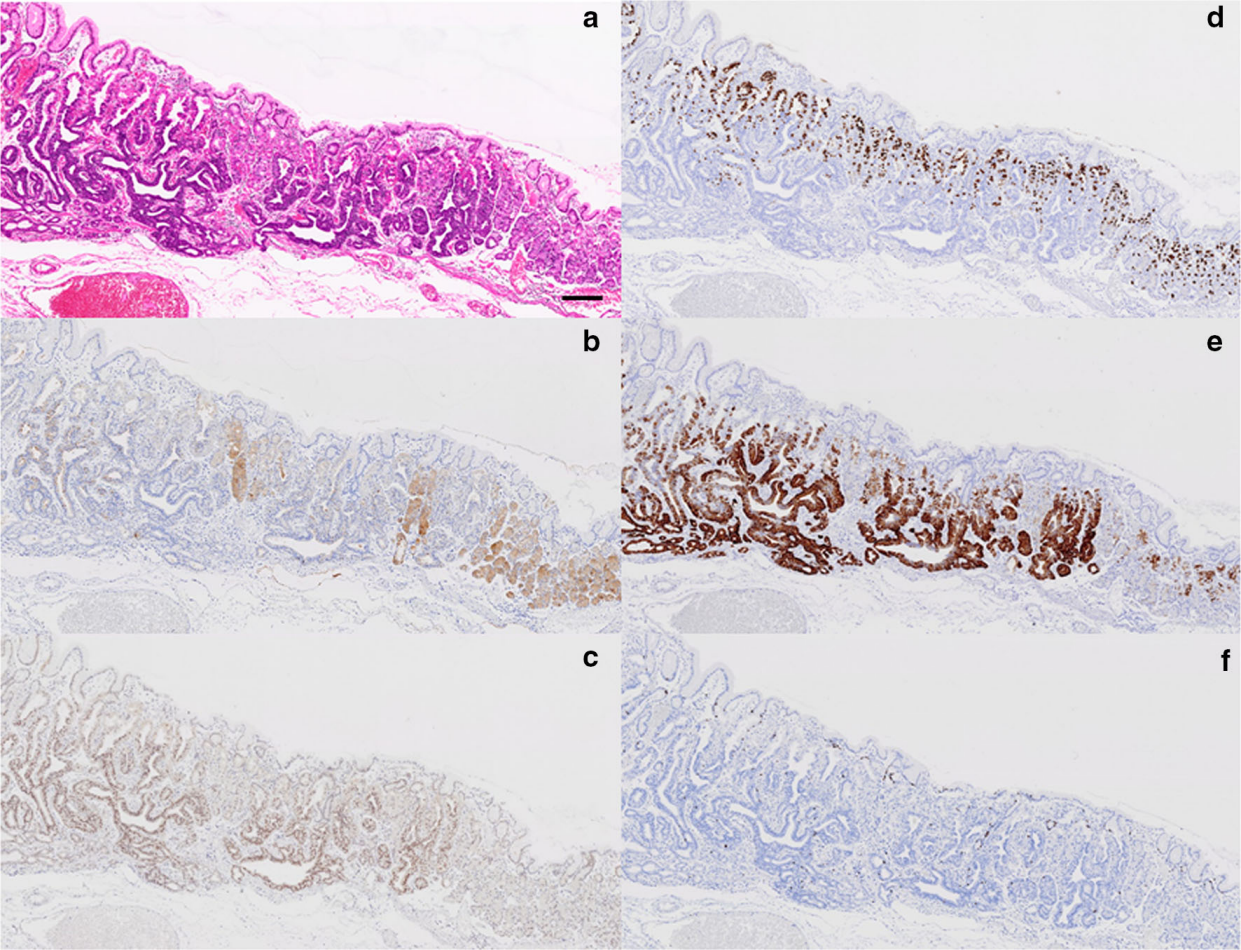
Histologic and immunohistochemical features of the adenocarcinoma and non-neoplastic fundic glands. **a** H&E stained tissue. The border between GAFG and non-neoplastic oxyntic mucosa is evident. **b** Pepsinogen-I staining. The neoplastic glands are more weakly stained than the non-neoplastic fundic glands. **c** Staining for MIST-1. Many positively stained cells are evident in the neoplastic glands. **d** Staining for H^+^/K^+^-ATPase. Positively stained neoplastic cells are prominent in the *upper* portion of the mucosa. **e** Staining for MUC6. The distribution of positively stained neoplastic cells corresponds well with that of cells stained positively for MIST-1. **f** Ki-67 staining. *Black bar* 200 μm

**Fig. 6:**
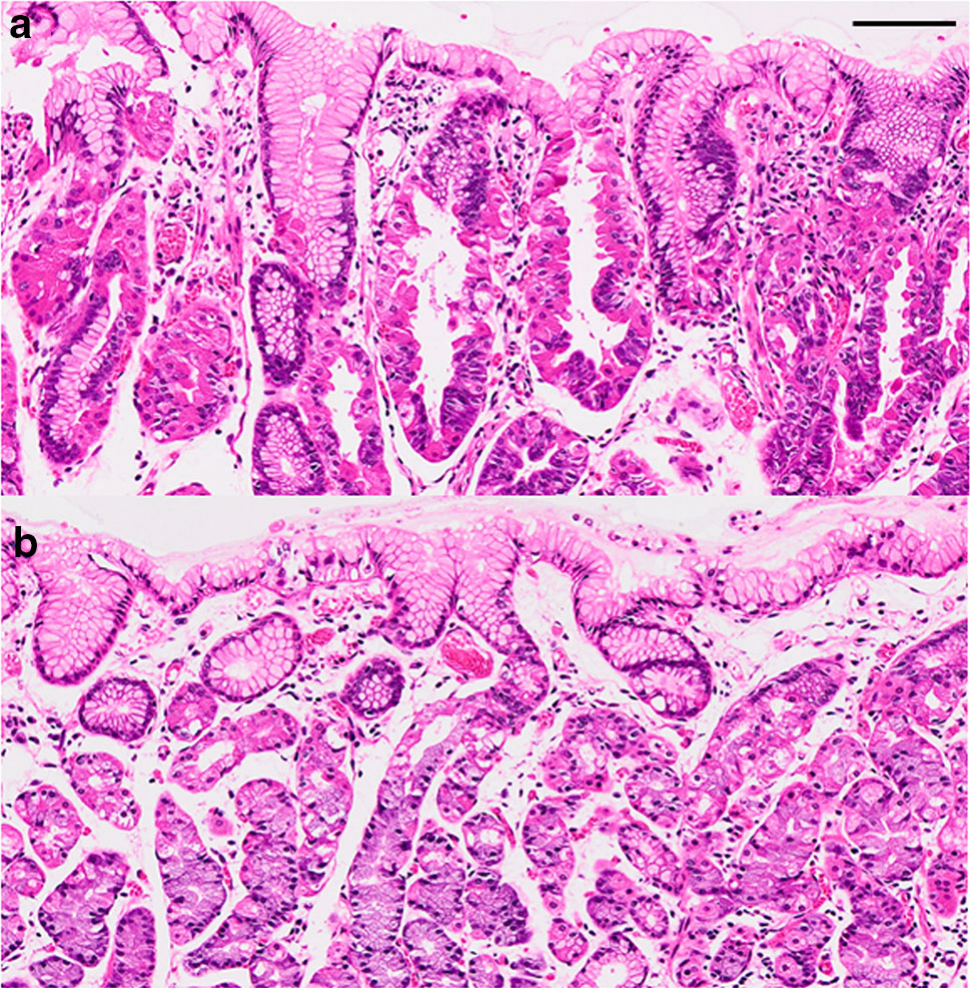
Histologic comparison of neoplastic glands and non-neoplastic fundic glands (hematoxylin and eosin-stained sections). **a** Protrusions are visible in the neoplastic cells showing not only parietal cell but also chief cell differentiation. **b** Protrusions are not seen in the non-neoplastic parietal cells. *Black bar* 100 μm

UGI endoscopy performed preoperatively confirmed the presence of an elevated, smooth, reddish, submucosal tumor-like lesion of ~10 mm located on the posterior wall of the fornix (Fig. 3a). The tumor was soft, and there was no evidence of expanding appearance. There was no clear line of demarcation, but dilated vessels with a branching architecture were apparent on the surface of the tumor. We observed a regular arrangement of collecting venules in the surrounding mucosa, and there were no signs of atrophy. Magnifying endoscopy with narrow band imaging (NBI) revealed dilatation of the intervening part between the crypts and microvessels without distinct irregularities (Fig. 3b). There was also no clear line of demarcation under NBI magnification. On the basis of the vessel plus surface (VS) classification system reported by Yao et al., a tumor with a regular microvascular (MV) pattern and regular microsurface (MS) pattern without a demarcation line was diagnosed [4]. After detailed examination, marks were placed around the tumor, and en bloc resection was achieved by ESD. The resected tissue measured 28 × 22 mm, and the flat elevated lesion measured 11 × 10 mm (Fig. 3c). Histopathologic features of the ESD specimen are shown in Fig. 4a–d. Histologically, the flat elevated lesion was formed by irregularly shaped glands composed of a mixture of slightly atypical glandular cells with basophilic cytoplasm and slightly atypical glandular cells composed of eosinophilic cytoplasm. These cells invaded the submucosa to approximately 300 µm. The surface of the lesion was covered by non-neoplastic foveolar epithelium. Immunohistochemical examination performed with antibodies to pepsinogen I, MIST-1, MUC6, and H^+^/K^+^-ATPase confirmed the fundic gland differentiation of the irregularly shaped glands. In addition, the Ki-67 labeling index (LI) was very low (Fig. 5a–f). These findings resulted in a diagnosis of GAFG (chief cell predominant type). Histopathological examination of the resected specimen also revealed protrusion of the neoplastic cells and dilation of the neoplastic glands (Fig. 6). The final post-ESD diagnosis was of a very well differentiated adenocarcinoma with chief cell differentiation. According to the Japanese classification of gastric carcinoma, the tumor was classified as follows: U, Post, type 0-IIa, tub2 > tub1, pT1b (SM), medullary type (med), INFa, ly0, v0, pHM0, pVM0. Curative resection under an expanded indication was achieved. Four years have passed since the surgery, and there has been no evidence of recurrence.

## Discussion

Gastric cancer with fundic gland differentiation, particularly of chief cells, was first reported by Tsukamoto et al. in 2007 [5]. In 2010, Ueyama et al. examined the clinicopathologic characteristics of numerous gastric carcinomas with chief cell differentiation and discovered a subset of tumors with mild cytologic atypia and fundic gland differentiation. Their study gave rise to a new disease concept: GAFG [1, 2]. GAFG is a type of gastric cancer that arises in the upper third of the stomach, the fundus, where tubular glands lie in the mucosa. It is not accompanied by atrophic gastritis related to *H. pylori* infection or intestinal metaplasia, and it accounts for just 1.6 % of all cases of gastric cancer [6].

In a retrospective study aimed at characterizing the endoscopic features of GAFG, Ueyama et al. found that six (60 %) of the ten GAFGs included had submucosal tumor-like protrusions, 40 % were flat or depressed, 70 % had a whitish appearance, and approximately half had dilated vessels with branching architecture [7]. Findings in our case were typical, including *H. pylori*-negative fundic gland mucosa in the upper third of the stomach, a submucosal tumor-like appearance, and dilated vessels with branching architecture on the lesion surface. Furthermore, black pigmentation was seen on the surface of the lesion 8 years after its initial endoscopic discovery, and such black pigmentation has been reported as one of the endoscopic features of GAFG [8]. According to the NBI magnification and VS classification systems, which are considered useful in the diagnoses of early gastric cancers [4], the microvascular/mucosal surface pattern was regular, and no particular carcinoma characteristics were detected. GAFG arises from the deep layer of the gastric mucosa, the lamina propria, and spreads laterally; the tumor itself is covered by normal epithelium and is not exposed to the surface layer. This seems to explain the macroscopic submucosal tumor-like appearance and the non-carcinoma-like NBI magnification features.

Our case emerged as an unusual opportunity for us to observe the natural history of GAFG over a 12-year period. The lesion was judged to be benign at the time of the initial UGI endoscopic examination. However, when, at 12 years, we reviewed the first biopsy specimen (Fig. 1b), we found that the GAFG was present at the time of the first UGI endoscopic examination. We believe that we overlooked the true nature of the lesion because the concept of GAFG did not exist at the time.

Fujisaki et al. followed the natural history of a normally differentiated gastric adenocarcinoma; the intramucosal cancer progressed to the advanced stage (muscularis propria invasion) over a period of 6 years [9]. Somewhat to the contrary, we observed no morphologic change in our patient’s tumor over a 12-year observation follow-up period; the tumor remained in the submucosal layer and was readily resected by ESD. No destruction of the muscularis mucosa was seen. Rather, the invading tumor split the submucosal layer, and no desmoplastic changes were observed. In addition, no lymph node or venous invasion was found. Progression of the disease clearly differed from that of a normal differentiating gastric adenocarcinoma as well as that of normal gastric cancer that infiltrates the submucosal layer.

We reviewed reports of GAFG published in the English and Japanese literature by 2015 and identified 48 cases, the details of which are shown in Table 1. Submucosal invasion was observed in 30 cases (62.5 %); mean lesion size was relatively small at 17.8 mm. Despite the high rate of submucosal invasion, no metastasis or recurrence was noted for any of the 28 submucosal lesions that were followed up (mean follow-up period: 19.1 months). The Ki-67 LI in our case was very low, and Yao et al. reported Ki-67 LIs that ranged from 1 to 15 % (mean: 5.6 %) for GAFG [2]. Thus, GAFG seems to have a low level of proliferative activity and minimal atypia. Singhi et al. have suggested that GAFG be reconsidered as an adenoma based on its low grade malignancy [12]. However, because some cases of advanced GAFG have been reported [3] and some GAFGs have exhibited vascular invasion, we believe that GAFG cases should be regarded as cancer cases. However, the time required for these tumors to develop into advanced cancers remains unclear, and further detailed investigations involving greater numbers of patients are needed to determine whether most cases can be treated in the same way as our patient’s GAFG was treated and whether any GAFGs exhibit high grade malignancy.

**Table 1:** Reports of gastric cancer of fundic gland type, 2011–2015

Authors	No. of patients	Age (years)	Sex (M/F)	Tumor location^a^	Tumor morphology	Tumor size (mm)	Invasion depth^b^	Ly+/v+	Follow-up time (months)	Recurrence
Ueo et al. [3]	1	62	1/0	M	0-IIc	44	SS	1/2	30	None
Ueyama et al. [10]	23	66.5 (51–78)	15/8	U16/M6/L1	^c^	12	M/6/SM17	2/0	29.1 (2–75)	None
Miyazawa et al. [11]	5	72.2 (67–78)	3/2	U5	^d^	15	SM5	1/0	10.6 (2–20)	None
Singhi et al. [12]	10	64.2 (44–79)	4/6	U10	0-I 10	4.9	M10	0/0	15 (6–39)	None
Park et al. [13]	3	65.3 (47–76)	3/0	U1/M1/L1	0-IIa + IIc 3	26.3	M1/SM2	0/0	24.3 (11–32)	None
Kato et al. [14]	1	80	1/0	U	0-IIa	30	SM	0/0	3	None
Fukatsu et al. [15]	1	56	1/0	U	0-IIa	5	SM	0/0	12	None
Yahata et al. [16]	1	47	1/0	M	0-IIc	18	SM	0/1	–	–
Fujimoto et al. [17]	1	72	1/0	U	0-IIa	15	SM	0/0	12	None
Miyaoka et al. [18]	1	59	0/1	U	0-IIb	8	SM	0/0	17	None
Fujisawa et al. [19]	1	50	1/0	M	0-IIc	40	SM	0/0	–	–

Number of patients is shown unless otherwise indicated

Mean (and range) values are shown when the number of patients is greater than 1

– Not reported

^a^Tumor location is shown as U, M, L (upper, middle, lower third of the stomach) with the corresponding number of patients

^b^Invasion depth is shown as M, SM, SS (mucosa, submucosa, subserosa) with the corresponding number of patients

^c^SMT (*n* = 13), 0-I (*n* = 1), 0-IIa (*n* = 2), 0-IIb (*n* = 1), 0-IIc (*n* = 6)

^d^0-IIa (*n* = 4), 0-IIb (*n* = 1)

Histopathological examination of the resected specimen in our case also revealed protrusion of the neoplastic cells and dilation of the neoplastic glands. The protrusions indeed resembled parietal cell protrusions (PCPs), which are now well-known histopathologic changes related to the use of proton pump inhibitors (PPIs) [20]. Interestingly, the protrusions were observed not only in the neoplastic cells showing parietal cell differentiation but also in those showing chief cell differentiation. Considering the fact that our patient had never been given PPIs, the carcinogenesis in our case remains undefined. Of note is the fact that both parietal cells and chief cells express aquaporin 4 (AQP4), namely, a water channel protein [21], and there has been recent speculation that an excessive influx of water resulting from an increase in the number of AQP4-positiveparietal cells gives rise to the formation of PCPs and cystic dilation of fundic glands [22]. Perhaps such a mechanism was responsible for the protrusions of neoplastic cells and dilation of neoplastic glands in our case. Further studies are needed for clarification of the underlying mechanism.

In conclusion, our long-term observation of the natural history of a GAFG confirm previous characterization of GAFG as a type of gastric cancer of low-grade malignancy. However, we have learned that GAFG can turn into a progressive cancer, and we anticipate further studies that will pinpoint the endoscopic and histopathologic features of GAFGs useful for evaluating the degree of tumor malignancy.
